# Small Heat Shock Protein αA-Crystallin Prevents Photoreceptor Degeneration in Experimental Autoimmune Uveitis

**DOI:** 10.1371/journal.pone.0033582

**Published:** 2012-03-30

**Authors:** Narsing A. Rao, Sindhu Saraswathy, Geeta Pararajasegaram, Suraj P. Bhat

**Affiliations:** 1 Doheny Eye Institute, University of Southern California, Los Angeles, California, United States of America; 2 Jules Stein Eye Institute, Brain Research Institute and Molecular Biology Institute, University of California Los Angeles, Los Angeles, United States of America; Istituto Superiore di Sanità, Italy

## Abstract

The small heat shock protein, αA-crystallin null (αA−/−) mice are known to be more prone to retinal degeneration than the wild type mice in Experimental Autoimmune Uveoretinitis (EAU). In this report we demonstrate that intravenous administration of αA preserves retinal architecture and prevents photoreceptor damage in EAU. Interestingly, only αA and not αB-crystallin (αB), a closely related small heat shock protein works, pointing to molecular specificity in the observed retinal protection. The possible involvement of αA in retinal protection through immune modulation is corroborated by adaptive transfer experiments, (employing αA−/− and wild type mice with EAU as donors and Rag2−/− as the recipient mice), which indicate that αA protects against the autoimmune challenge by modulating the systemic B and T cell immunity. We show that αA administration causes marked reduction in Th1 cytokines (TNF-α, IL-12 and IFN-γ), both in the retina and in the spleen; notably, IL-17 was only reduced in the retina suggesting local intervention. Importantly, expression of Toll-like receptors and their associated adaptors is also inhibited suggesting that αA protection, against photoreceptor loss in EAU, is associated with systemic suppression of both the adaptive and innate immune responses.

## Introduction

Autoimmune Uveoretinitis leads to irreversible loss of vision due to degeneration of the photoreceptor neurons in the retina. The photoreceptor degeneration in uveitis is seen as a culmination of the inflammatory cascade that involves T-cell mediated adaptive immune response [1], [2], [3]. The rodent EAU model [4] presents a clinically relevant paradigm for a phenotypic assessment of various gene products whose expression attends the inflammatory cascade that leads to blindness through degeneration of photoreceptor neurons. In mice availability of various genetic knockouts provides additional tools for the assessment of the relevance of specific gene products in inflammation as well as in understanding their possible utility in therapeutic intervention.

Among the gene products that are differentially expressed in EAU in mice the small heat shock protein, αA-crystallin (αA) shows significantly elevated expression in the inner segments of photoreceptor neurons, the site of oxidative damage in the retina. This increased expression of αA was associated with the protection of photoreceptor neurons in the retina [5]. At the cellular level these data fit well into known anti-apoptotic activity of this small heat shock protein in cultured cells [6], however it does not illuminate the relationship of αA, if any, to the immune status of the animal and/or the pathology of the eye with autoimmune retinitis. In this report using αA null (−/−) [7] and wild type (WT) mice we have examined the effect of intravenous administration of αA on retinal integrity and investigated the status of various immune modulators associated with the protection of photoreceptor neurons in EAU.

## Results and Discussion

Small heat shock proteins have been suggested to be neuro-protective [8]. Masilmoni et al., first reported that intraperitoneal administration of α-crystallin (a mixture of two small heat shock proteins, αA and αB proteins) affords protection against silver nitrate –induced inflammation in rats [9], [10], [11]. Interestingly, intravenous injections of αB in mice have been shown to ameliorate the MS symptoms [12]. αB is the major gene product expressed at high levels in multiple sclerosis [13] and other neurodegenerative disease including Alzheimer's disease [14]. The αB gene (*Cryab*) contains a canonical heat shock promoter [15] and therefore much against the prevailing status of the expression of stress proteins and their association with neurodegenerative diseases [8], [14], it was surprising to find that in EAU, in mice, it was not αB that showed increased expression but it's very close relative, the αA [5].

Autoimmune Uveitis is a disease that is restricted to the visual system, but its management has focused mostly on systemic suppression of the immune system responses [16]. In view of this background we investigated the effect of intravenous administration of αA protein on retinal integrity and photoreceptor degeneration in αA null (−/−) and wild type (WT) mice upon immune challenge in EAU. We also followed the attendant immune response(s) by assessing the relevant gene activity encompassing cytokines and innate immune response.

**Figure 1:**
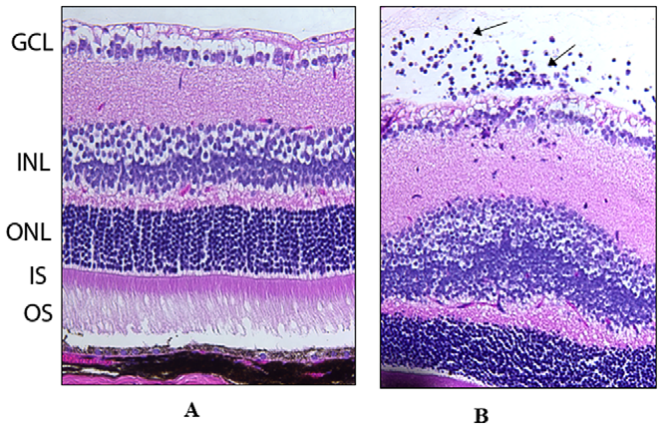
αA administration protects retinal phenotype in αA−/− mice in EAU. Prevention of inflammation by intravenous administration of αA crystalline protein in αA −/− mice with EAU on day 14 post immunization (A) whereas in the untreated αA −/− mice with EAU (B), there was inflammatory cell infiltration (arrows) and retinal damage. GCL = Ganglion cell layer, INL = Inner nuclear layer, ONL = Outer nuclear layer, IS = inner segments, OS = outer segments. Magnification: 400×.

**Figure 2:**
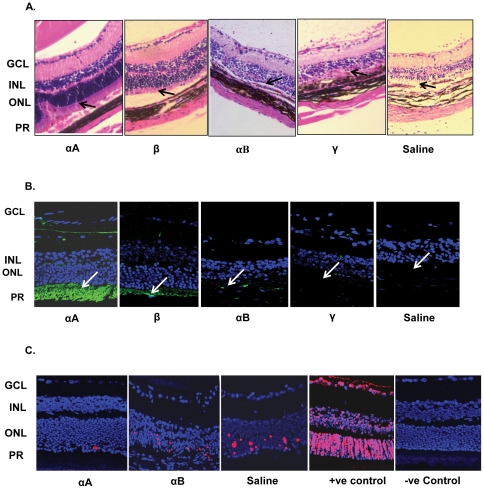
Intravenous administration of αA protects retinal photoreceptors. Twelve days after EAU was induced in B10RIII (WT) mice with an IRBP peptide, the animals were injected with various crystallins and the retinas were collected and examined on day 21. **A, B and C:** αA = αA crystallin, β = β crystallin, αB = αB crystallin, γ = γ crystallin and saline. **A.** Histology of retina and uvea (Hematoxylin and Eosin stained). Arrows indicate photoreceptor inner segments (PR). Note presence of photoreceptors in αA and their degeneration (absence) in Saline treated animals. **B**. Retinal architecture as revealed by immunostaining for IRBP with anti-IRBP (green, arrows). **C.** Apoptosis in EAU retinas of animals treated with various crystallins; αA treated, αB treated, saline treated, positive (+ve) control (DNase I treated retina) and (‘no enzyme’) negative (−ve) control. Note that retinal photoreceptors are preserved (**A,** αA and **B,** αA); there is little or no apoptosis (TUNEL positive cells) in αA treated animals (**C,** αA). PR = Photoreceptors, ONL = Outer nuclear layer, INL = Inner nuclear layer, GCL = Ganglion cell layer.

Intravenous administration of αA selectively protects retina in αA−/− mice against autoimmune induced inflammation and cell death in EAU (Fig. 1). This remarkable protection of the retinal phenotype in αA−/− EAU mice points to the importance of this small heat shock protein in the maintenance of the immune health in the mouse. This protection against inflammatory incursions and retinal degeneration is also evident in WT type mice. Well-preserved photoreceptors are seen in WT mice with EAU treated with αA (Fig. 2A and B). Among other crystallins, only β-crystallin treated animals showed some visible protection (Fig. 2A and B, compare αA with β-crystallins, αB and γ and saline control). In αA treated EAU animals retinal integrity is maintained as corroborated by the measurement of retinal thicknesses, which were significantly higher in αA treated than in αB, γ-crystallin and saline treated animals (p<0.001) (see Table S1). Notably, these retinas also showed absence of apoptotic cells (Fig. 2C) in animals treated with αA, an observation that is in accord with the known anti- apoptotic properties [6] and increased expression αA in the EAU retina [5].

It is important to note that the photoreceptor protection is protein-specific (Fig. 2). Out of the two α-crystallins it is only αA, which protects retina. This data is highly significant because it points to existence of differential modes of mechanistic intervention by two closely related small heat shock proteins. αA and αB are highly conserved small heat shock proteins, which share 58% sequence similarity (for review see [14]). Additionally, it is interesting to note that among these two α-crystallins (αA and αB) only αA is detected in the spleen and thymus, while both of them are expressed in the retina [17], [18]. Both of these small heat shock proteins have been shown to be anti-apoptotic [6], however, it is only αA that is elevated in EAU [5]. It is, therefore, noteworthy that high levels of αA, expressed in the retina during the inflammatory response, are localized to the inner segments of the photoreceptor neurons where pathological oxidative stress predominates in EAU [5], [19].

In considering the induction of the expression of the two α-crystallins (αA and αB) it is important to consider that these two gene products are differentially regulated. The promoter architecture of the two genes is different [14]. It is the αB gene that contains a canonical heat shock promoter [15] and would have been expected to be induced under apparent “pathological stress” yet it is the expression of αA gene, which lacks a recognizable stress promoter that is elevated. These observations suggest a non-conventional and physiologically-specific mechanism of stress protein regulation in EAU.

**Figure 3:**
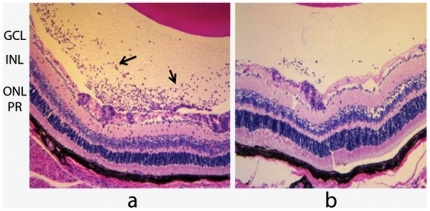
Adaptive transfer demonstrates involvement of αA with immune regulation. Inflammatory cell infiltration is a hallmark of the progression in EAU. Note inflammatory cells (arrows) in the vitreous in (a) representing the status of early retina in Rag2−/− mice injected with splenocytes and lymphocytes from αA−/− mice with EAU. Note the relative absence of this infiltration in b, which represents the status of retina in Rag2−/− mice injected with splenocytes and lymphocytes derived from the wild type mice (129Sve) with EAU. PR = Photoreceptors, ONL = Outer nuclear layer, INL = Inner nuclear layer, GCL = Ganglion Cell layer.

In order to investigate the role of αA in photoreceptor protection and its possible relationship to immune mechanisms operative in EAU we conducted adaptive transfer experiments employing the αA null [7] and Rag2−/− mice (devoid of T and B cells). EAU was induced in αA−/− mice with Interphotoreceptor Retinol-binding protein (IRBP) peptide and reactive splenocytes and lymphocytes from these mice where then injected into Rag2 −/− mice. Infiltration of inflammatory cells was seen in the retinas of Rag2 −/− mice on day eleven after transfer (Fig. 3), a feature that is characteristic of early autoimmune uveitis [4], [5]. This transfer eventually leads to severe retinal degeneration in Rag2−/− mice compared to the retina of Rag2−/− mice transferred with the splenocytes and lymphocytes derived from wild type (WT) mice with EAU (Fig. 3). These data indicate that absence of αA increases the effectiveness of reactive splenocytes and lymphocytes in this paradigm, which essentially tests the potency of the adaptive immune network. As indicated above, EAU in rodents is known to be a T cell-dependent immune process that involves Th1 and/or TH17 effectors [1], [4], [20] but this does not preclude the requirement of a robust initial innate immune response [1]. It is possible that αA presence modifies the initial innate immune response that may impact the ensuing adaptive immune response that has an immunosuppressive phenotype as initially reported for hsp10 [21].

**Figure 4:**
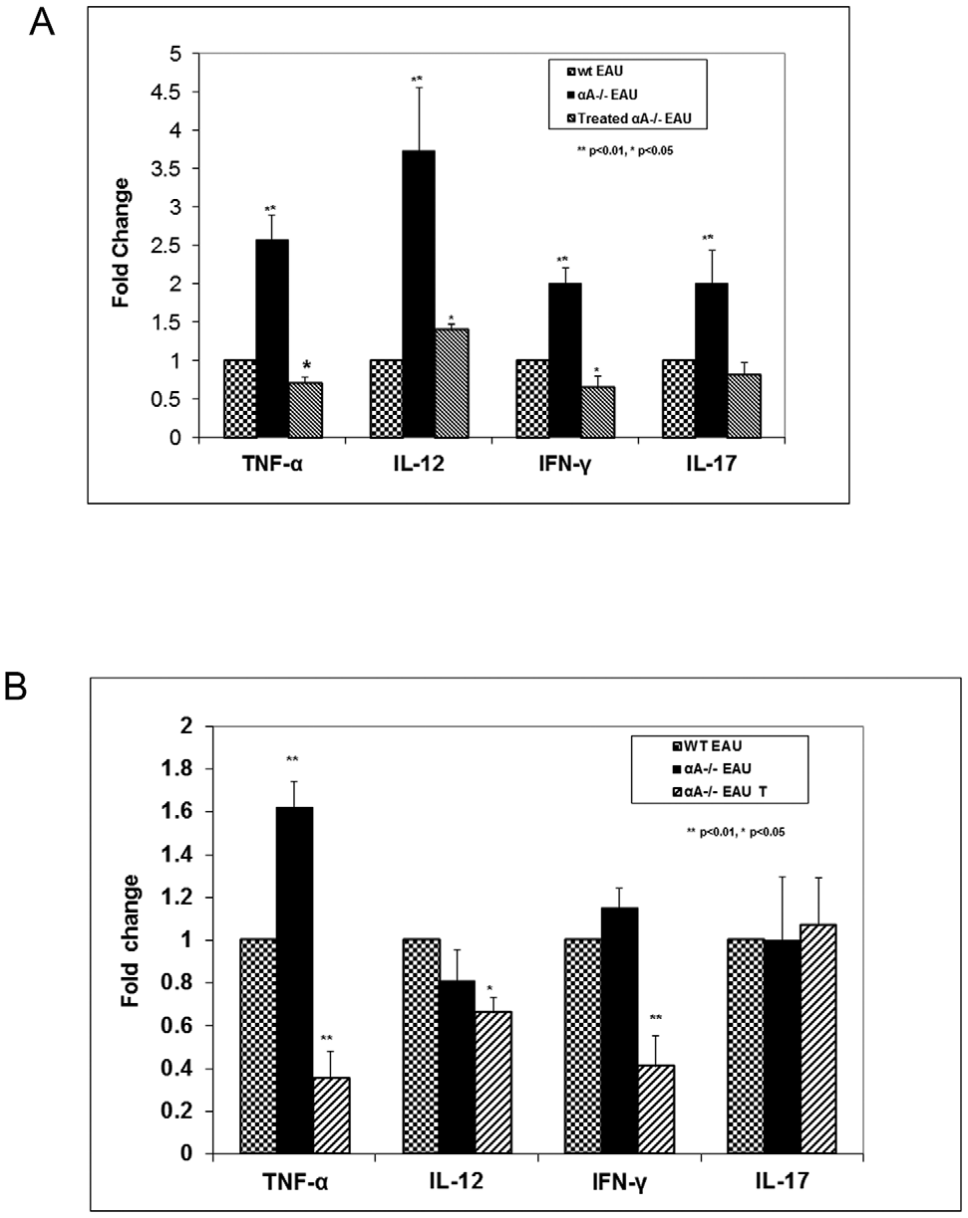
αA treatment has a significant impact on cytokine expression. **A** and **B** are RT-PCR assays (A). Retinal Th1 cytokines (TNF-α, IL-12 and IFN-γ) and TH17 cytokine (IL-17) are up-regulated on day 21 in αA−/− EAU mice compared to the WT EAU controls after a challenge with the IRBP peptide. With αA administration (treated αA−/− EAU) these cytokines show significantly reduced expression compared to the untreated αA−/− EAU mice. (B). In the spleen of αA−/− with EAU, the levels of TNF-α show an increase compared to the WT mice with EAU. IL-12 and IFN-γ expression is similar in knockout mice and the WT mice. αA administration reduces the levels of TNF-α, IL-12 and IFN - γ in the spleen in αA−/−mice compared to the untreated αA−/−mice. Note that IL-17 remains unchanged in all three groups of mice. This is in contrast to increased expression of IL-17 in the retinas of αA −/− EAU mice and its inhibition in animals treated with αA (see A).

**Figure 5:**
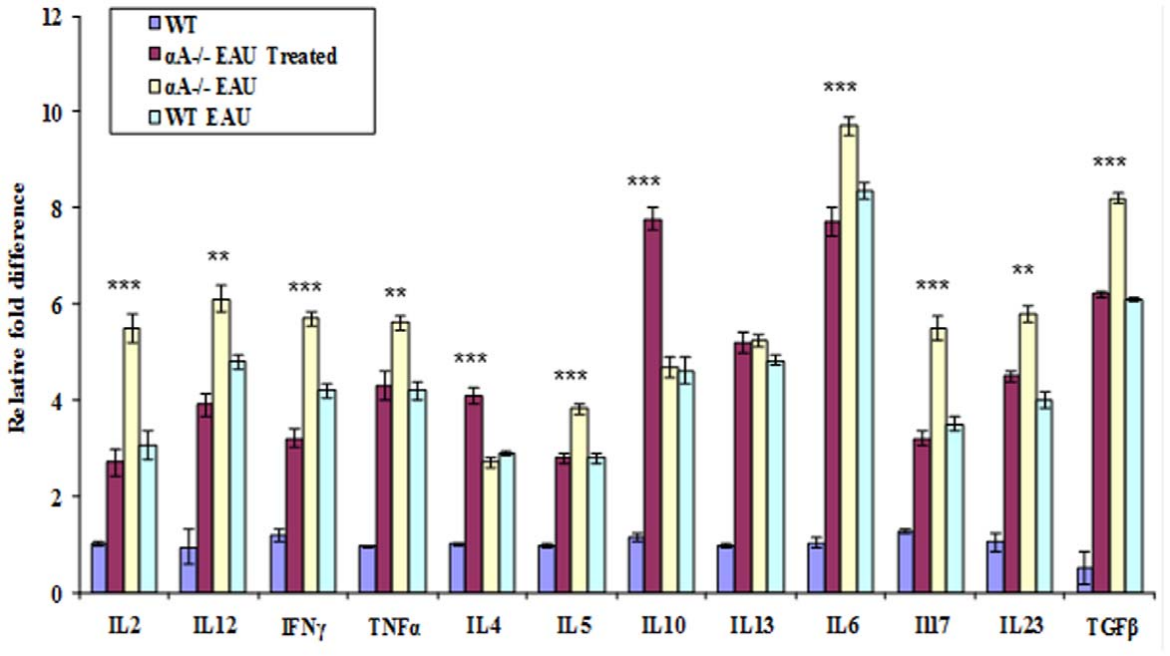
αA administration markedly lowers protein levels for various cytokines. ELISA assays show decreased levels of IL-2, IL-12, IFN-γ and TNF-α and Th17 cytokines IL-6, IL-17, IL-23 and TGF-β in EAU mice treated with αA, while Th2 cytokines IL-4 and IL-10 levels are increased.

The innate immune response that culminates in T cell-mediated inflammatory process is heralded when bacterial components present in CFA are recognized by Toll like receptors (TLRs) [22], [23], [24]. We, therefore, evaluated cytokine gene expression in spleen and retina in the context of our investigation in wild type (WT) mice with EAU, αA−/− mice with EAU and αA−/− mice with EAU treated with intravenous αA. We have previously shown that αA−/− EAU mice in comparison to WT EAU mice show early inflammatory response and apoptosis in the retina and present with significant inflammation in the uvea on day 18 after immunization [5]. There are early signs of inflammatory cell infiltration in αA−/− mice with EAU but not in the retina of αA−/− mice treated with αA (see Fig. 1) RT-PCR analyses of the gene expression of various cytokines in the retina and the spleen is even more informative. Intravenous administration of αA neutralized all induced cytokine gene expression (TNF- α, IL-12 and IFN γ), both locally (in the retina) as well as in the spleen (Fig. 4, A and B and Table S2). Multi analyte ELISA array analysis showed that the Th1 cytokines IL-2, IL-12, IFN-γ and TNF-α were significantly down-regulated in the retina upon αA protein administration to αA−/− mice with EAU (Fig. 5 and Table S3).

IL17 is known to play a role in the severity of inflammation in EAU [25]. The concentrations of Th17 cytokines IL-6, Il-17, Il-23 and TGF-β were also lowered in αA treated mice (retinas), whereas the Th2 cytokines, Il-4 and IL-10 were elevated (Fig. 5). While it is interesting to note the elevation of IL12, a cytokine involved in enhancing TH1 responses [26], the data on IL17 stands out; its expression was elevated only in the retina of the αA−/− mice; there was no significant change in expression in the spleen of these mice (Fig. 3B) and corresponding WT animals with EAU (compare Figures 4A and 4B, IL-17). Importantly, administration of αA to αA−/− EAU mice inhibits IL17 increase in the retinas of these animals (Fig. 4A). These observations prompt the conclusion that αA administration may impact the retina in a more specific fashion where marked elevation of this cytokine (IL17) occurs during early phase of EAU development [25]. This conclusion is supported by observations that IL-17 −/− mice show known reduced inflammation in EAU [27] and the recent report on the amelioration of the severity of EAU by all-trans retinoic acid, which inhibits the emergence of the IL17 producing Th17 cells [28]. In light of this data an investigation into a molecular mechanistic link direct or indirect (via Th17 cells) between αA and IL17 expression will be rewarding.

**Figure 6:**
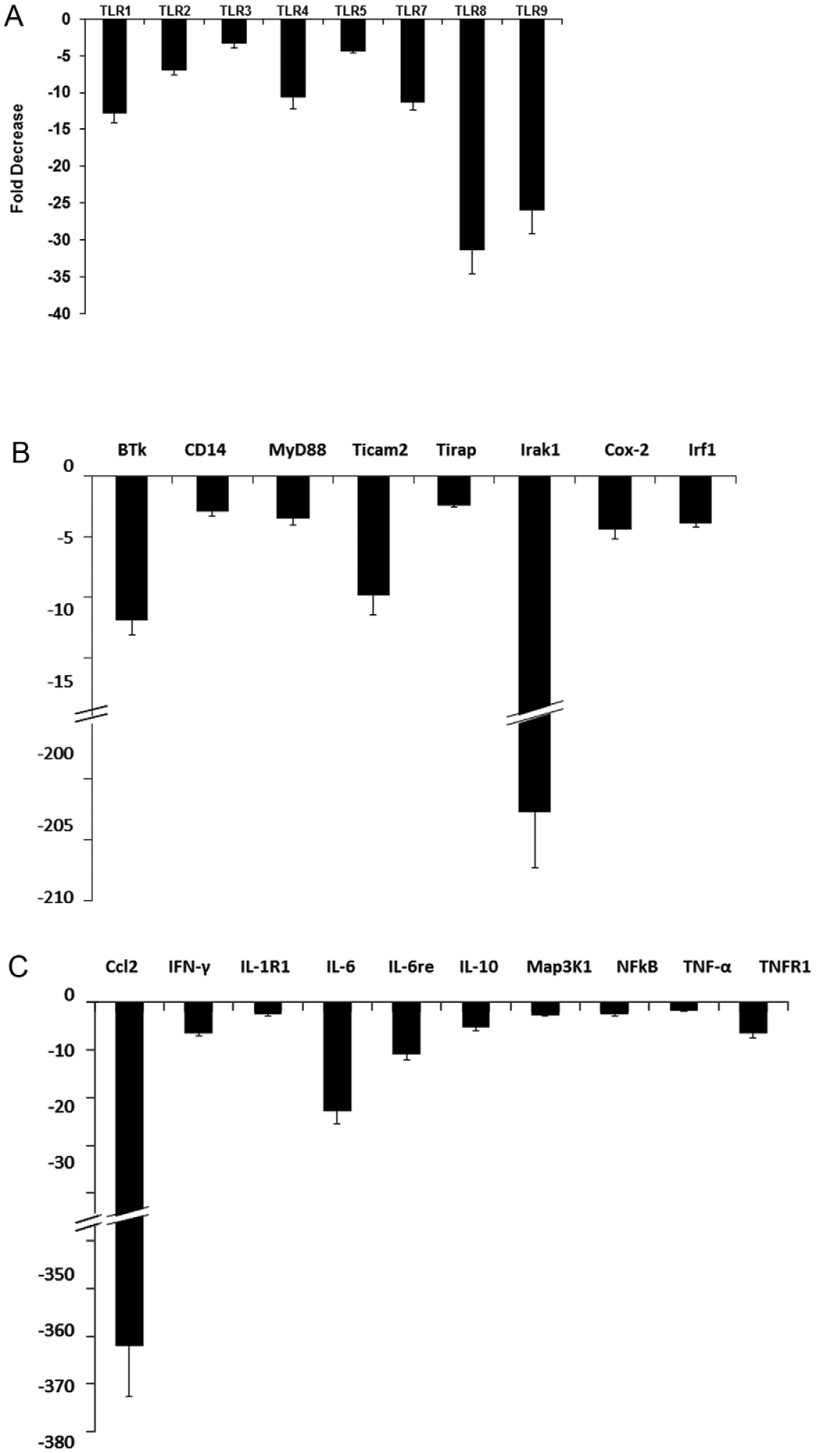
Down regulation of gene activity associated with the innate immune pathways. Commercial PCR arrays were used. Fold decrease was calculated by comparing data obtained with αA−/− EAU without αA treatment and αA−/− EAU mice treated with αA. A = Toll like receptors B = associated adaptive and signaling proteins and C = the NFkB pathway in αA−/− mice with EAU treated with αA. TLRs = Toll like receptors, MyD88: Myeloid differentiation primary response gene (88), Irak1 = Interleukin-1 receptor-associated kinase 1, CD14 = Cluster of differentiation 14, Ticam 2 = TIR domain-containing adapter molecule 2, Tirap = toll-interleukin 1 receptor (TIR) domain containing adaptor protein, Cox-2 = Cyclooxygenase-2, Irf-1 = Interferon regulatory factor 1, IL-1R1 = Interleukin 1 receptor, type I, Il-6re = IL6 responsive element, Map3K1 = Mitogen-activated protein kinase kinase kinase 1, NFkB = Nuclear factor kappa B, Ccl2 = Chemokine (C-C motif) ligand 2, IFN-γ = Interferon-gamma.

**Figure 7:**
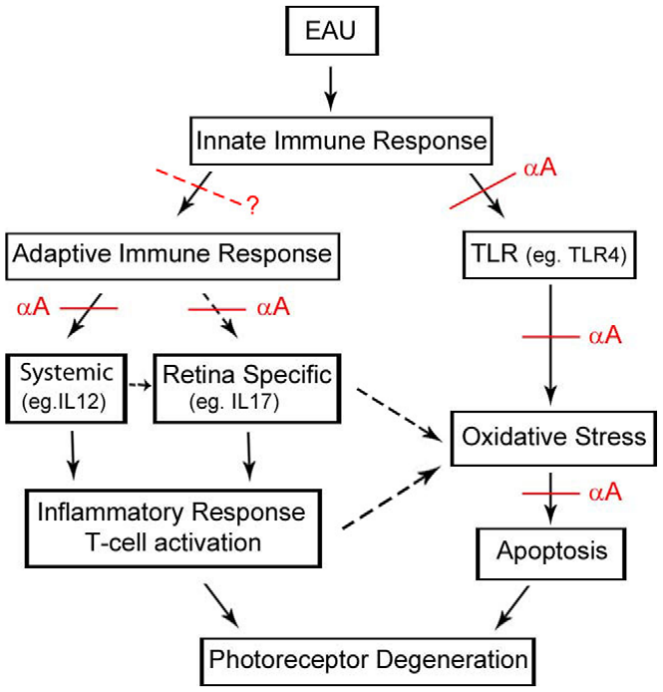
A schematic showing possible pathways of αA intervention that lead to the protection of the neuroretina in EAU. These points of intervention have been deduced from the data presented in this manuscript and in the literature as discussed in the text. The oxidative stress in the mitochondria could have a common origin in the initial innate immune response as well as via the adaptive immune pathways. On the other hand this could also come from the pathological stress exacerbated by the adaptive immune system with systemic and retina-specific inputs (these possibilities have been indicated by a dotted black arrows), although the relationship between the two responses remains to be elucidated. The red dotted line with a question mark represents a speculation about the inhibition of the initiation of the adaptive immune response(s) after the initial activation of the innate immunity.

We next evaluated the impact of αA administration on the expression of Toll-like receptors (TLRs) and their associated adaptor gene products. We used commercially available PCR based arrays of genes involved in innate immune response (Fig. 6). αA administration results in 3 to 31 fold down regulation in TLRs 1–9; adaptors MyD88, Irak1, CD14, Tirap; and NFkB related genes including Ccl2, IFN- γ, NFkB, TNF-α and TNF-α receptors. This systemic down regulation suggests a response that may lead to a dampening of the innate immune response [3], [29], [30], [31] (Fig. 6). In the context of increased αA expression, the modulation of the TLRs and their adapter proteins may have multiple consequences beyond their significant role in the initiation of adaptive immune response. For example TLR4 may have a role in initiating mitochondrial oxidative stress [20], [32], [33]. Thus inhibition of TLR4 expression upon αA administration may negate increase of mitochondrial oxidative stress that may in turn contribute to photoreceptor protection (see Fig. 7), which connects with the observation of enhanced αA in photoreceptor neurons. It is also important to note that αA has been reported to be an endogenous ligand for TLR4 [34], which may also have important roles in regulation of the progenitor cell proliferation in the retina [35].

Taken together the observations detailed above on the protection of the retinal photoreceptor neurons by αA administration in EAU point to functions of this small heat shock protein in immune regulation, beyond its anti-apoptotic properties [5], [6]. Based on the data presented here and published literature (as discussed above) we have summarized possible αA intervention points (Fig. 7). It is apparent that small heat shock proteins may follow specific routes or pathways in modulating the immune activities such as their specific role in immunosuppression [36], and for example αB has been characterized as the target for the adaptive immune system [37]. The data in this manuscript demonstrates that intravenous administration of αA to mice with EAU results in highly specific down regulation of innate as well as adaptive immune pathways, which seem to be organ specific (retina) suggesting that this small heat shock protein may prove of potential therapeutic benefit in the protection of photoreceptor neurons in uveitis and related T cell-mediated immune retina/uvea dysfunctions.

## Materials and Methods

Animal care and use was in compliance with institutional guidelines and with the Association for Research in Vision and Ophthalmology Statement for the Use of Animals in Ophthalmic and Vision Research. αA and αB were recombinant full length human proteins purchased from Abcam (San Francisco, CA). β-crystallin and γ-crystallin fractions prepared from the bovine lens were purchased from Stressgen Bioreagents.

### Treatment of EAU animals with crystallins

EAU was induced in 8-week-old B10.RIII mice (Jackson Laboratory, Bar Harbor, ME) by subcutaneous injections of complete Freund's adjuvant containing Interphotoreceptor Retinol-binding Protein (IRBP) peptide [5]. On day 12 post injection (pi), the animals received 10 ug of recombinant αA or 10 ug of recombinant αB or 10 ug of β-crystallin or γ-crystallin in saline intravenously on alternate days. The mice were sacrificed on day 21, enucleated eyes were processed for histologic and immunohistochemical staining using rabbit anti-IRBP antibody (1∶50) and Alexa-Fluor488-conjugated goat anti-rabbit IgG (1∶200). Isotype controls and primary antibody replaced by PBS were used as the negative controls. The experiments were performed in triplicate. Retinal thickness at the juxtapapillary, equator and ora serrata areas was measured and analyzed by one way analysis of variance (ANOVA) using Tukey Kramer multiple comparisons test (data in Table S1).

Twelve αA−/− mice were similarly immunized with an IRBP peptide [1] and divided in to two groups (six each). One group received 10 ug of recombinant αA on alternate days starting from day 7 post immunization and the control group received saline. On day 14 after immunization, the mice were sacrificed and the eyes analyzed histologically. The experiments were performed in triplicate (three groups of six animals each).

### Apoptosis by TUNEL Assay

On day 21 post immunization, retinas from B10RIII EAU mice (Six each) treated with αA, or αB or saline were assayed for apoptosis employing the TUNEL assay (Roche Diagnostics, IN). DNAse 1 (Roche Diagnostics) treated tissue was used as the positive control and ‘no enzyme’ as the negative control. Staining was performed in triplicate.

### Adaptive transfer

αA−/− and WT (129Sve) mice were immunized with IRBP peptide [32]. The lymph nodes and spleen cells were isolated at day 10 post-injection (pi) as described [4] (Caspi, 2003). They were then pooled and cultured in the presence of 10 µg of IRBP peptide for 3 days. The cells were transferred into new flasks every day to remove adherent monocytes and macrophages. The cells were collected and washed. Live cells (10×10^6^) were injected intraperitonealy into 6 week old Rag2−/− mice (Taconic Farms). The mice were also injected with 5 ug of pertussis toxin intraperitoneally. On day 11 post transfer the mice were sacrificed and the eyes were studied histologically. The experiments were performed in triplicate.

### Th1 and Th17 cytokines gene expression in αA crystallin treated EAU animals

Eighteen αA −/− mice and six WT (129Sve) mice were immunized with IRBP peptide as above. Out of these, from day 12 onwards, a group of six mice were intravenously injected with 10 ug of recombinant αA, a second group of six with 10 µg of recombinant αB and a third group of six mice with saline, on alternate days. The mice were sacrificed on day 21; retinas were dissected out for RNA extraction (TriZol reagent, Invitrogen). The cDNAs were generated (Omniscript RT kit; Qiagen, Valencia, CA) and PCR was conducted with gene specific primers for TNF-alpha, IL-12, IFN gamma and IL-17 using the I-cycler (BioRad). Glyceraldehyde-3-phosphate dehydrogenase (GAPDH) was used as the normalizing gene as it did not show any significant changes in our experimental protocols. The PCR reactions for each gene in each experiment were performed in triplicate on each cDNA template, along with triplicate reactions for the housekeeping gene GAPDH. The threshold cycle (Ct) difference between the experimental and control groups, for each gene in each tissue, was calculated and normalized to GAPDH, and the increase (*x*-fold) in mRNA expression was determined by the 2-ΔΔct method. Statistical analysis of ΔΔCt was performed with a Student's *t*-test for three independent samples, with significance set as *P*<0.05, and compared between the different experimental groups.

### Determination of cytokines Th1 and Th17 protein in αA crystallin treated EAU animals

Eighteen αA−/− mice and WT (129Sve) mice were immunized with IRBP peptide as above and injected intravenously with 10 ug of recombinant αA or αB or saline from day 12 onwards on alternate days. The protein levels of the cytokines, Th1, Th2, Th17 from the retinas were determined by ELISA using a Multi-Analyte ELISArray kit (SA Biosciences) according to manufacturer's instructions. Actual amount of protein in each assay is given in Table S3.

### Assessment of the expression of Toll like receptors and their signaling pathway genes in αA treated EAU animals

A group of 18 wild type mice and two groups of 18 αA−/− mice were immunized with IRBP peptide as described above. Out of the two groups of αA−/− one group of 18 was injected intravanously with recombinant αA from the day before immunization and all three groups of animals were sacrificed on day 7. The retinas were dissected for RNA extraction by the TriZol method (Invitrogen, Carlsbad, CA). RNA was quantified and checked for purity and integrity by microanalysis in an Agilent Bioanalyzer (Agilent Technologies, Santa Clara, CA). These RNAs were used to prepare cDNAs, which was used for PCR array analyses (SABiosciences, MD) for Toll like receptors and its signaling genes including adapters, interacting proteins and NFkB signaling genes. All the three experimental groups were compared to each other. The experiments were run in triplicate.

## Supporting Information

Table S1 **Morphometric analysis of the retinas after treatment with crystallins.** Morphometric analysis of retinal thickness in microns from the Juxtapapillary, Equator and Ora Serrata areas of EAU mice treated with αA, αB, β and Gamma crystallins and saline.
(DOCX)

Table S2 **Cycle threshold values from qPCR analysis on mice retinas after alpha A crystalline treatment.** This table provides Cycle threshold values (an average of three determinations) for the data (fold changes in expression) shown in Figures 3A and 3B. mRNA was isolated from the retina and spleen. WT EAU = Wild type mice with EAU, αA−/− EAU = αA knockout mice with EAU, Treated αA−/− EAU = αA−/− mice with EAU treated with αA intravenous injections. Details are given under Materials and Methods.
(DOCX)

Table S3 **Analysis of protein levels of Th1/Th2/Th17 cytokines by ELISA.** Data showing the cytokine levels in pg/ml in the retina of wild type mice (WT), WT mice with EAU (WT EAU), αA −/− mice with EAU (αA−/− EAU) and, αA −/− mice with EAU treated with αA protein (Treated αA−/− EAU). Multi- Multi-Analyte ELIS Array Kit (Qiagen) analyzes a panel of 12 cytokines involved in T helper cell biology using a conventional ELISA protocol all at once under uniform conditions. For the ELISA assay, we used 12 retinas (6 mice) as one sample. The protein was extracted from these samples and from the total 600 ul of the protein; 50 ul of the protein sample (40 ug) was added to each well containing each cytokine. This was done in triplicate.(See Materials and Methods for details).
(DOCX)
