# Supplementary material for: Small Heat Shock Protein αA-Crystallin Prevents Photoreceptor Degeneration in Experimental Autoimmune Uveitis
Source: PLoS One. 2012 Mar 30;7(3):e33582. doi: 10.1371/journal.pone.0033582 (PMC3316578; doi:10.1371/journal.pone.0033582)
Supplement: Table S1 — Morphometric analysis of the retinas after treatment with crystallins. Morphometric analysis of retinal thickness in microns from the Juxtapapillary, Equator and Ora Serrata areas of EAU mice treated with αA, αB, β and Gamma crystallins and saline. (DOCX) [file pone.0033582.s001.docx]

**Supplementary Table S1.**

| **Treatment groups** | **Juxta papillary area(µ)** | | **Equator area (µ)** | | **Ora Serrata area (µ)** | |
| --- | --- | --- | --- | --- | --- | --- |
| **EAU with Saline** | Mean ± s.e.m  141.90± 9.53 | Median  141.05 | Mean± s.e.m  119.24±6.72 | Median  119.33 | Mean± s.e.m  86.15±2.63 | Median  86.18 |
| **EAU with αA crystallin** | 195.42±5.82 | 203.20 | 179.09±9.36 | 181.74 | 112.64±6.76 | 108.20 |
| **EAU with αB crystallin** | 146.88±8.44 | 150.11 | 108.16±5.72 | 108.19 | 80.08±3.22 | 92.22 |
| **EAU with β crystallin** | 192.11±10.58 | 201.55 | 165.47±10.23 | 170.51 | 107.20±5.75 | 108.84 |
| **EAU with γ crystallin** | 146.78± 9.92 | 138.17 | 119.99±6.33 | 122.56 | 87.64±3.32 | 90.30 |

s.e.m = standard error of mean
