# Supplementary material for: Small Heat Shock Protein αA-Crystallin Prevents Photoreceptor Degeneration in Experimental Autoimmune Uveitis
Source: PLoS One. 2012 Mar 30;7(3):e33582. doi: 10.1371/journal.pone.0033582 (PMC3316578; doi:10.1371/journal.pone.0033582)
Supplement: Table S2 — Cycle threshold values from qPCR analysis on mice retinas after alpha A crystalline treatment. This table provides Cycle threshold values (an average of three determinations) for the data (fold changes in expression) shown in Figures 3A and 3B. mRNA was isolated from the retina and spleen. WT EAU = Wild type mice with EAU, αA−/− EAU = αA knockout mice with EAU, Treated αA−/− EAU = αA−/− mice with EAU treated with αA intravenous injections. Details are given under Materials and Methods. (DOCX) [file pone.0033582.s002.docx]

**Supplementary Table S2**.

| Cytokines | Groups of mice | CT values of retina | CT values of spleen |
| --- | --- | --- | --- |
| TNF alpha | WT EAU | 33.5 | 32.8 |
|  | αA-/- EAU | 33.34 | 31.00 |
|  | Treated αA-/- EAU | 35.00 | 33.20 |
|  |  |  |  |
| IL12 | WT EAU | 33.00 | 29.00 |
|  | αA-/- EAU | 32.30 | 29.3 |
|  | Treated αA-/- EAU | 33.50 | 30.10 |
|  |  |  |  |
| IFNg | WT EAU | 30.3 | 28.00 |
|  | αA-/- EAU | 30.5 | 28.3 |
|  | Treated αA-/- EAU | 31.9 | 29.80 |
|  |  |  |  |
| IL-17 | WT EAU | 28.00 | 28.4 |
|  | αA-/- EAU | 26.3 | 28.2 |
|  | Treated αA-/- EAU | 27.5 | 28 |
