# Supplementary material for: Small Heat Shock Protein αA-Crystallin Prevents Photoreceptor Degeneration in Experimental Autoimmune Uveitis
Source: PLoS One. 2012 Mar 30;7(3):e33582. doi: 10.1371/journal.pone.0033582 (PMC3316578; doi:10.1371/journal.pone.0033582)
Supplement: Table S3 — Analysis of protein levels of Th1/Th2/Th17 cytokines by ELISA. Data showing the cytokine levels in pg/ml in the retina of wild type mice (WT), WT mice with EAU (WT EAU), αA −/− mice with EAU (αA−/− EAU) and, αA −/− mice with EAU treated with αA protein (Treated αA−/− EAU). Multi- Multi-Analyte ELIS Array Kit (Qiagen) analyzes a panel of 12 cytokines involved in T helper cell biology using a conventional ELISA protocol all at once under uniform conditions. For the ELISA assay, we used 12 retinas (6 mice) as one sample. The protein was extracted from these samples and from the total 600 ul of the protein; 50 ul of the protein sample (40 ug) was added to each well containing each cytokine. This was done in triplicate.(See Materials and Methods for details). (DOCX) [file pone.0033582.s003.docx]

**Supplementary Table S3.**

| **Cytokines** | **αA-/- EAU Treated** | **αA-/- EAU** | **WT EAU** | **WT** |
| --- | --- | --- | --- | --- |
| IL2 | 2.7 | 5.5 | 3.06 | 1.02 |
| IL12 | 3.9 | 6.1 | 4.8 | 0.94 |
| IFN γ | 3.2 | 5.7 | 4.2 | 1.2 |
| TNF α | 4.3 | 5.6 | 4.2 | 0.95 |
| IL4 | 4.1 | 2.7 | 2.9 | 1 |
| IL5 | 2.8 | 3.82 | 2.8 | 0.966 |
| IL10 | 7.77 | 4.7 | 4.6 | 1.14 |
| IL13 | 5.2 | 5.24 | 4.82 | 0.96 |
| IL6 | 7.7 | 9.7 | 8.36 | 1.02 |
| IL17 | 3.2 | 5.5 | 3.5 | 1.26 |
| IL23 | 4.5 | 5.8 | 4 | 1.04 |
| TGF β | 6.2 | 8.2 | 6.1 | 0.51 |
